# Healing the health system after civil unrest

**DOI:** 10.3402/gha.v8.27381

**Published:** 2015-03-30

**Authors:** Somali-Swedish Action Group for Health Research and Development

**Keywords:** action, Diaspora, health systems, research collaboration, war and conflicts

Over the last quarter century, the Somali population has endured protracted internal conflicts with devastating effects on the delivery of essential and lifesaving health care services. This extended humanitarian crisis situation has seriously weakened the public health sector, causing high maternal and child mortality; heavy burden of communicable and non-communicable diseases, including mental disorders; and emergency levels of malnutrition. The need to increase the delivery of equitable, affordable, and sustainable health care services to the population is a huge challenge to health sector recovery initiatives. Academic institutions have important roles in responding to the existing health workforce crisis as well as in carrying out and building capacity for research to guide health sector development activities.

To address these issues a seminar was organised on 2–3 December 2014 by Umeå University, Sweden, in collaboration with the Somali-Swedish Researchers’ Association (SSRA), a small Swedish NGO. The 53 participants, who included representatives of national Somali and Swedish universities and agencies, as well as health professionals from the Somali Diaspora, shared an overwhelming commitment to forge collaborative action for Somali health research and development. At the end of the two days of deliberations, the participants agreed on a joint statement, committing themselves to work for national and international partnerships in support of efforts to revitalise the Somali health systems and to promote and strengthen capacity for research as a key component in health development.

The aim of publishing this statement is to raise awareness among and promote a response by the international community to address the formidable challenges and pressing unmet needs facing the rehabilitation and recovery of the health sector in post-conflict situations. The aim is also to draw attention to the need for integrating health research into these efforts in order to provide evidence for the design of sector policies and intervention programmes. Lessons learnt from the Somali situation may be of great value to guide health sector development after civil unrest in other settings – now and in the future.

## Statement by seminar participants

Based on our fundamental recognition of health as a human right, we shared information about ongoing efforts to rebuild the Somali health systems and identified the needs and opportunities for national and international collaborative partnerships. Recognising the value of a former programme of research cooperation sponsored by Sweden in the 1980s and early 1990s, special focus was given to the role of national academic institutions in promoting health development and sustainable health services. Renewed activities aimed at strengthening the capacity of Somali institutions for training and research, in cooperation with Swedish agencies and institutions as well as with the Somali Diaspora, were explored.

We noted the ongoing efforts and determination to extend essential health services to all Somali communities, while remaining cognisant of the many constraints and challenges facing them, which include:

- The lack of a critical mass of trained staff, inadequate infrastructure, and shortage of financial resources at all levels of the health care systems, as well as for academic institutions;
- The need to address the glaring health and nutrition problems of mothers and children, which also demonstrate the need for reliable community-based, especially longitudinal, data to set priorities and evaluate programmes;
- The urgent need to provide high quality health services, including essential medicines and vaccines, and, while adopting a gender perspective, to give high priority to the elimination of harmful traditional practices like female genital cutting (also referred to as female genital mutilation), which are the cause of much suffering;
- The inadequate attention being paid to the social determinants of health, which are essential in efforts to achieve universal access to basic primary education, gender equity, provision of safe water and sanitation, and the safeguarding of human security and development;
- The striking lack of coordination, due to political and safety concerns, which limits the outreach and efficiency of both the health services and university systems;
- Inadequate legislation, regulatory functions, and accreditation systems with adverse effects on health services as well as academic work;
- The need to apply modern communication techniques in health research as well as service delivery.

With this background, we affirmed our commitment to the following:

### Health services for all

- All levels in the Somali health systems, and all associated policies, need to be developed and supported so that they are accountable, of high quality, and well regulated.
- Enhanced and continuing education for all health workers, managers, and administrators as well as scaled–up leadership capacities are central pre-requisites for an effective health care system, and should be prioritised.
- Among other key concerns, the health services should focus on reproductive, maternal and child health, mental disorders, and communicable diseases, and they should be delivered and managed by well-trained health professionals, including a strong cadre of female community health workers.
- The direction of the health services should be guided by sound evidence derived from operational and evaluative research, which in turn should be based on a comprehensive situational analysis of service delivery needs.

### Community participation and ownership

- It is critical that the voice of the Somali people is taken into account in the provision of universally accessible and acceptable health services. Priorities should be based on perceived health needs of the community, which could be identified using social and anthropological research methods. The particular needs of neglected and vulnerable populations – such as pregnant women, children under the age of five, people with mental disorders, and the disabled – should be in focus.
- The social determinants of health, particularly water and sanitation, security, food, and education, should be investigated, as should health-seeking behaviours and community health financing. It is only through such efforts that the health services will be ‘owned’ by the community, a key prerequisite for their effectiveness and sustainability.
- The training of traditional birth attendants, community health workers (in particular women), and managers, is essential, and should be guided by lessons learnt from other post-conflict settings.

### Academic institutions as key actors

- The links between health research, policy, and practice need to be actively nurtured. The respective actors and stakeholders in each of these spheres must work together to ensure the provision of high quality, evidence-based health services that meet the needs of the people.
- Sweden’s support to the Somali health sector, which is mainly channelled through the UN Joint Health and Nutrition Programme, could be complemented by the Swedish International Development Cooperation Agency (Sida) defining Somalia as a priority country for research cooperation in order to create a knowledge base for policy development and forge sustainable links between policy and development programmes in the health sector.
- In order to ensure a consistent and high quality of medical and other health professional training in the country, the educational curricula in all the Somali institutions providing such training must be harmonised. The Somali Research and Education Network (Somali-Ren) should take the lead in organising the required mapping and coordination of all the stakeholders (local as well as foreign). All academic institutions involved in training health professionals should be accredited by recognised government regulatory bodies as well as relevant Somali education and health authorities.
- Development of academic research capacity is required, from bachelor’s through to postgraduate level. A new postgraduate sandwich training programme between Somali and Swedish academic institutions would support this process.
- Universities should engage in vocational and midlevel health professional training, so that the health needs of the community are addressed.
- A comprehensive mapping of specific research needs (which could include learning from other post-conflict settings) is required, and the establishment of a health and demographic surveillance system would provide an excellent platform for such research over the longer term.
- Sustainable research collaborations need to be built in direct support of health service delivery, with the involvement of international partners and members of the Somali Diaspora, based on long-term funding commitments. Special efforts are needed to recruit and sponsor talented and experienced Diaspora individuals for various Somali academic and public health posts and functions.

We, the participants of this meeting, commit ourselves to work for the promotion of national and international partnerships in support of Somali health development, and to keep the momentum in pursuing all the noble objectives delineated above towards that end. We pledge to promote health research as a key component of the national rebuilding process, to bridge the gap between knowledge and action in the country, and to contribute to developing the Somali primary health care system based on the principles of universal and equitable access to health and health care.

**Table:** 

List of participants/signatories		

Category	Name	Affiliation
Organisers	Anneli Ivarsson	Unit of Epidemiology and Global Health, Umeå University
	John Kinsman	Unit of Epidemiology and Global Health, Umeå University
	Karin Johansson	Unit of Epidemiology and Global Health, Umeå University
	Khalif Bile Mohamud	Somali-Swedish Researchers’ Association (SSRA)
	Lars Weinehall	Unit of Epidemiology and Global Health, Umeå University
	Lennart Freij	Somali-Swedish Researchers’ Association (SSRA)
	Stig Wall	Unit of Epidemiology and Global Health, Umeå University
Somali in-country	Abdirisak Ahmed Dalmar	Benadir University, Mogadishu
representatives	Abdirashid Omer Ibrahim Abdisamad Abikar Hagi	Dental School, Amoud University, Somaliland Benadir University, Mogadishu
	Abshir Ali Abdi	Faculty of Medicine, East Africa University, Bosasso, Puntland
	Abdullahi Sheik Hussein	Benadir University Foundation, Mogadishu
	Abdulkadir Mohamed Shirwa	Medical College, Galkayo University
	Amina Warsame	Network Against Female Genital Mutilation in Somaliland (NAFIS), Hargeisa, Somaliland
	Derie Ismail Ereg	Medical College, Hargeisa University, Somaliland
	Mohamed Hussain Aden	Medical College, University of Science and Technology, Puntland
	Maryan Qasim	Prime Minister’s Office, Federal Government of Somalia, Mogadishu
	Mohamed Khalid Ali	Faculty of Medicine, East Africa University, Puntland
Somalis in the ‘Diaspora’	Abdullahi Elmi Abdullahi Warsame Afrah	Royal Institute of Technology, Stockholm Scandinavian Health Care Ltd, Borlänge, Sweden
	Faduma Omar Sabtiye	Regional Hospital, Örebro, Sweden
	Fatuma Ege Guled	Tallbohov’s Nursing Home, Stockholm/SSRA
	Hinda Jama Ahmed	WHO Regional Office, Cairo, Egypt/SSRA
	Halima Mohamed	London School of Hygiene & Tropical Medicine, UK
	Halima Ali Tinay	Centre for Dependency Disorders, Stockholm County Council/SSRA
	Kadigia Ali Mohamud	University of Rome, Italy
	Mariam Warsame Yusuf	Global Malaria Programme, WHO, Geneva, Switzerland/SSRA
	Mayeh Omar Yakoub Aden Abdi	Nuffield Centre for International Health and Development, University of Leeds, UK Centre for Dependency Disorders, Stockholm County Council/SSRA
	Yusuf Abdulkadir	Somali-Swedish Researchers’ Association (SSRA)
Swedish university	Annika Johansson	Department of Public Health Sciences, Karolinska Institutet, Stockholm/SSRA
representatives	Asli Ali Kulane	Department of Public Health Sciences, Karolinska Institutet, Stockholm
	Barbara Schumann	Unit of Epidemiology and Global Health, Umeå University
	Birgitta Essén	Department of Women’s and Children’s Health, Uppsala University
	Faustine Nkulu Kalengayi	Unit of Epidemiology and Global Health, Umeå University
	Fredrik Elgh	Department of Clinical Microbiology, Umeå University
	Fredrik Norström	Unit of Epidemiology and Global Health, Umeå University
	Göran Lönnberg	Unit of Epidemiology and Global Health, Umeå University
	Helene Norder	Department of Clinical Microbiology, Sahlgrenska University Hospital, Göteborg
	Julia Schröders	Unit of Epidemiology and Global Health, Umeå University
	Kerstin Erlandsson	Dalarna University, Falun – Borlänge
	Kerstin Edin	Unit of Epidemiology and Global Health, Umeå University
	Klas-Göran Sahlén	Unit of Epidemiology and Global Health, Umeå University
	Lars L Gustafsson	Department of Laboratory Medicine, Karolinska Institutet, Stockholm
	Lars-Åke Persson	Department of Women’s and Children’s Health, Uppsala University
	Malin Eriksson	Unit of Epidemiology and Global Health, Umeå University
	Maria Emmelin	Department of Social Medicine and Global Health, Lund University
	Marie Hasselberg	Department of Public Health Sciences, Karolinska Institutet, Stockholm
	Marie Klingberg	Dalarna University, Falun – Borlänge
	Raman Preet	Unit of Epidemiology and Global Health, Umeå University
	Ulf Högberg	Department of Women’s and Children’s Health, Uppsala University
Swedish agencies	Urban Sjöström	Somalia Section, Swedish Embassy, Nairobi
	Saif Omar	Forum Syd, Stockholm

